# Facets of Religion/Spirituality and Cognitive Health: Association Variations Across Gender and Race Among Older Adults

**DOI:** 10.3390/rel16091204

**Published:** 2025-09-19

**Authors:** Katherine Carroll Britt, Augustine Cassis Obeng Boateng, Chinwe Nwadiogbu, Sato Ashida, Daniel Tranel, Roland J. Thorpe, Nabila Dahodwala

**Affiliations:** 1 College of Nursing, University of Iowa, Iowa City, IA 52242, USA; 2 School of Nursing, University of Pennsylvania, Philadelphia, PA 19104, USA; 3 Perelman School of Medicine, University of Pennsylvania, Philadelphia, PA 19104, USA; 4 College of Public Health, University of Iowa, Iowa City, IA 52242, USA; 5 Roy J. and Lucille A. Carver College of Medicine, University of Iowa, Iowa City, IA 52242, USA; 6 Bloomberg School of Public Health, Johns Hopkins University, Baltimore, MD 21205, USA

**Keywords:** Alzheimer, dementia, mild cognitive impairment, religious involvement

## Abstract

Religion and spirituality (R/S) may be associated with better cognitive health, yet most published studies have been conducted in primarily White populations without investigating association variations by gender and race. A cross-sectional analysis of 1,041 community-dwelling diverse older adults from the Philadelphia Healthy Brain Aging (PHBA) cohort study was conducted using multiple regression analysis. We examined associations between facets of R/S and total cognitive scores and performed stratification analysis separately by gender and race to explore potential gender- and race-specific variations. Higher non-organizational R/S was associated with lower cognitive scores, while greater religious and spiritual coping was associated with higher cognitive scores, controlling for age, education, chronic conditions, race, and financial constraints. Across gender and race variations, non-organizational R/S was associated with lower cognitive scores in women alone, with no variations across race. Higher religious and spiritual coping was associated with higher cognitive scores in both Black and White women, but not men, while higher religious and spiritual healing was associated with lower cognitive scores in Black women only. Associations between religious and spiritual facets and cognitive health differ across gender and race; longitudinal studies are needed.

## 1. Introduction

Cognitive disparities in neurodegenerative disease persist across race/ethnicity and gender. The prevalence of Mild Cognitive Impairment (MCI) and Alzheimer’s disease (AD) and Alzheimer’s disease related dementias (AD/ADRD) is approximately two times higher in Black Americans than in White Americans, largely due to persistent inequalities in social determinants of health (Alzheimer’s Association 2023). By 2060, minoritized ethno-racial groups will account for 45% of the U.S. population aged 65 years and older, prompting a projected increase in the prevalence of MCI to 4.45 million and AD/ADRD to 3.10 million in Black Americans (Rajan et al. 2021). Black Americans experience worse cognitive function, revealing racial/ethnic cognitive disparities (Babulal et al. 2019; Diaz-Venegas et al. 2018). In addition, with higher survival rates, women are at greater risk of MCI and AD/ADRD, with an estimated 4.2 million women living with AD/ADRD in the U.S. compared to 2.7 million men aged 65 years and older (Alzheimer’s Association 2024). Cognitive impairment drives substantial healthcare costs, including medical expenses and care, increasing the burden on care partners and placing them at twice the risk of emotional, physical, and financial problems (Alzheimer’s Association 2023, 2024). Currently, there is no cure for AD/ADRD, but many of the risk factors are modifiable lifestyle factors (Livingston et al. 2020), where we also see disparities. These lifestyle factors are modifiable (e.g., differential levels of exercise, diets among minoritized groups), making them a target to slow cognitive decline and reduce disparities.

A salient resource in communities that experience persistent social and health inequities, older Black adults report the importance of religion and spirituality (R/S) to cope with stress and illness (Chatters et al. 2015). Associated with better mental health outcomes, religious and spiritual practices are lifestyle factors that promote well-being (Koenig et al. 2023), which is reported to influence brain atrophy rates in regions affected by AD/ADRD (Farhang et al. 2019). Promoting resilience against brain changes (Abellaneda-Pérez et al. 2023), as well as meaning and purpose in life, which are supported through spirituality, is associated with slower rates of cognitive decline and a reduced risk of MCI and AD/ADRD (Bell et al. 2022; Boyle et al. 2010). Women in the U.S. are more likely than men to focus on R/S, engaging more in prayer, reading religious texts, and committing to their religious and spiritual community (Upenieks and Zhu 2024; Schnabel 2017). One study (Corsentino et al. 2009) found that for women with higher levels of depression, less frequency of religious and spiritual service attendance was associated with greater cognitive decline compared to higher frequency of religious and spiritual service attendance. This association did not hold for men; however, another study (Inzelberg et al. 2013) found that for women but not for men, prayer frequency was associated with a lower risk of MCI.

Studies suggest religious and spiritual practices may be associated with better cognitive function (Coin et al. 2010; Sauerteig-Rolston et al. 2024; Vitorino et al. 2023), including the domains of memory and attention (Lekhak et al. 2020; Luhrmann et al. 2013), and executive control (Basso et al. 2019). R/S may serve as a protective factor against cognitive decline, yet studies are limited to primarily White samples and fail to consistently explore potential gender differences across associations, which is important. Women tend to be more religious and spiritual than men, engaging in religious and spiritual activities such as prayer, worship, and reporting faith as very important to them; these differences appear to result from social differences across norms, roles, and expectations (Trzebiatowska and Bruce 2012). In addition, impacts may be stronger in Black older adults, considering a stronger emphasis on the importance of R/S for coping and a higher risk of MCI and AD/ADRD.

Modifiable protective factors need to be identified to support successful aging and slow decline in older adults at greater risk of MCI and AD/ADRD. Studies examining facets of R/S and cognitive health in diverse older adults will bring great public health benefits (Britt et al. 2023; Hosseini et al. 2019). Since R/S are multidimensional constructs, examining facets of R/S (e.g., specific religious and spiritual activities, beliefs) will help tease out specific targets for slowing cognitive decline and inform cognitive health lifestyle modification recommendations incorporating spirituality. In addition, a focus on understanding how R/S intersects with race and gender is vital to optimize strategies to slow cognitive decline, as women and historically underrepresented populations are at greater risk of AD/ADRD. Two recent studies identified variations in religious service attendance and cognition across race (Henderson et al. 2021; Sauerteig-Rolston et al. 2024), but only one explored variation across gender (Sauerteig-Rolston et al. 2024). While Henderson et al. (2021) focused on religious service attendance and religiosity, Sauerteig-Rolston et al. (2024) focused on religious service attendance and religious salience. Our study fills the gap to focus on unexplored facets of R/S that include non-organizational R/S, religious and spiritual coping, and religious and spiritual healing. Thus, this study aims to examine the association between facets of R/S and cognitive impairment among a diverse sample of older adults in the Philadelphia Greater Metropolitan area and elucidate variations across gender and race.

Therefore, this study addresses the following questions:

1. What is the association between specific facets of R/S, specifically, non-organizational R/S, religious and spiritual coping, and religious and spiritual healing and cognitive health in older adults?
2. Do these associations vary by gender and race?

We hypothesize that positive religious and spiritual coping will be associated with better cognitive health outcomes, and that these associations will differ across gender and racial groups.

## 2. Methods

### 2.1. Study Design & Population

This is a retrospective cross-sectional secondary analysis of community-dwelling, English-speaking, older adults from the Philadelphia Healthy Brain Aging (PHBA) study. Individuals in the PHBA study were recruited from four primary care clinics within the University of Pennsylvania Health System, located in Philadelphia, Pennsylvania, between January 2010 and December 2012.

### 2.2. Data Collection

Participants’ self-identified demographic information, including their race and ethnicity according to U.S. census-defined categories, age, concerns about contemporary financial status, and years of formal educational attainment. Every participant underwent cognitive assessment using the Montreal Cognitive Assessment (MoCA) administered by a trained research staff (Nasreddine et al. 2005).

### 2.3. Study Eligibility

Inclusion criteria for our study were older adults aged 55 years or older, English-speaking, and capable of completing the study questionnaire independently. Exclusion criteria included self-reported or health records confirmation of a prior diagnosis of Parkinson’s disease (PD), Alzheimer’s disease (AD), or dementia at baseline, or an incomplete MoCA.

### 2.4. Measures

#### 2.4.1. Cognition

The primary outcome measure was the MoCA score. The MoCA test includes assessment of visuospatial/executive, naming, memory, attention, language, abstraction, delayed recall, and orientation abilities (Nasreddine et al. 2005). MoCA scores range from 0 to 30, with a 1-point addition for individuals if the level of education is less than or equal to 12 years. Higher scores indicate better cognition. A score of less than 25 is suggestive of cognitive impairment even after adjustment. For the analyses in the current study, the total MoCA score of each participant was used.

#### 2.4.2. Religiosity/Spirituality

R/S were assessed using the Lukwago Religiosity Scale, a validated instrument with religious and spiritual items of behaviors and beliefs (Lukwago et al. 2001). The instrument includes four Likert-scale items assessing religious and spiritual beliefs (e.g., “I am often aware of the presence of God in my life”) and five items about religious and spiritual behaviors (e.g., “I often read religious books, magazines, or pamphlets”). A composite score was calculated by summing the responses to individual items on the scale, with higher scores reflecting greater religious commitment or involvement. To examine various facets of R/S more closely, religious and spiritual items were divided into three categories: nonorganizational religiosity, religious coping, and religious healing. Nonorganizational R/S was measured by a composite score based on religious and spiritual prayer and reading, while religious and spiritual coping was assessed through a composite score incorporating the presence of God, a personal relationship with God, and spirituality. Religious and spiritual healing was measured using a single item that asked participants if they felt healed through religious and spiritual activities.

Non-organizational R/S:

- I often read religious books, magazines, and pamphlets
- I pray often

Religious and spiritual coping:

- I am often aware of the presence of God in my life
- I have a personal relationship with God
- My spiritual beliefs are the foundation of my whole approach to life

Religious and spiritual healing:

- When I am ill, I pray for healing

### 2.5. Statistical Analysis

First, we conducted bivariate analysis for each independent variable and the main outcome to assess their relationships. The association between three facets of R/S (e.g., non-organizational R/S, religious and spiritual coping, and religious and spiritual healing) and cognitive function was examined using multiple regression analysis, controlling for age, education, chronic conditions, race, and financial constraints. Multiple regression was performed after a univariate analysis using the equation ***y* = *b***_**1**_***×***_**1**_ **+ *b***_**2**_***×***_**2**_ **+ … + *b***_***n***_***x***_***n***_ **+ *c***, (Cohen et al. 2002) where ***bi’s* (*i* = 1, 2… *n*)** are the regression coefficients, which represent the value at which MoCA score changes when R/S (***x***_**1**_) change holding other covariates constant (***x***_**2**_**-----*x***_***n***_). A complete case analysis (listwise deletion) was employed, resulting in a sample size reduction from 1052 to 1041.

To account for potential gender-specific and race influences, we stratified our analysis separately by gender and then by race, enabling a more nuanced understanding of how religious and spiritual facets influence cognition scores in men and women, followed by Black and White participants. We used multivariable regression analysis with 95% confidence intervals (CIs). All statistical tests were 2-sided using *p* < 0.05 for significance and performed in Stata 18.

## 3. Results

### 3.1. Descriptive Statistics

Table 1 presents descriptive statistics for a sample of 1041 participants. The mean MoCA total score was 24.997 (*SD* = 3.991). A majority fraction of the participants was Black (55%) and women (55.9%), with a mean age of 68.554 years (*SD* = 8.985). Participants had at least some money left over after paying for essentials (62.2%), while 24.6% had just enough, and 13.2% did not have enough money left after paying for essentials.

### 3.2. Regression Analyses

Table 2 displays the results of regression models examining predictors of total MoCA scores. In the unadjusted model (Model 1), higher non-organizational R/S was associated with lower total MoCA scores (b = −0.240, *p* < 0.001), while greater religious and spiritual coping was associated with higher MoCA scores (b = 0.402, *p* < 0.001). Religious and spiritual healing was not significantly associated with total MoCA scores in the unadjusted model.

After adjusting for demographic and health covariates (Model 2), the relationships between non-organizational R/S (b = −0.153, *p* < 0.05) and religious and spiritual coping (b = 0.278, *p* < 0.001) with total MoCA scores remained significant, while religious and spiritual healing was still not significantly associated with total MoCA score. Being Black (b = −2.333, *p* < 0.001) and older age (b = −0.099, *p* < 0.001) were associated with lower total MoCA score. Education level (b = 0.714, *p* < 0.001) and having some money left (b = 0.846, *p* < 0.05) were positively associated with total MoCA score.

In Table 3 across gender, the relationship between R/S facets and total MoCA score shows the analysis separately in women and men, revealing association differences across gender. All three R/S variables were significantly associated with MoCA in women alone: higher nonorganizational R/S and religious and spiritual healing were both significantly associated with lower MoCA scores (b = 10.171, *p* = 0.026; b = −0.320, *p* = 0.020) while higher religious and spiritual coping was associated with higher MoCA score (b = 0.454, *p* < 0.000) in women alone. There were no significant R/S associations with MoCA in men.

In Table 4 across race, the relationship between R/S variables and total MoCA score shows the analysis separately in Black and White adults. Associations also differed by race as higher religious and spiritual coping was associated with higher MoCA scores (b = 0.443, *p* < 0.000) while higher religious and spiritual healing (b = −0.393, *p* = 0.019) was associated with lower MoCA scores in Black adults; only higher religious and spiritual coping was significantly associated with higher MoCA scores in White adults (b = 0.226, *p* = 0.008).

## 4. Discussion

The objective of our study was to explore associations of various facets of R/S with cognitive health in diverse older adults, examining variations across gender and race. Our analysis found significant associations between religious and spiritual facets (i.e., non-organizational R/S, religious and spiritual coping) and cognitive function among older adults in Philadelphia, Pennsylvania, U.S., with significant patterns holding only in women but not men; in addition, variations were seen across Black and White adults, too. Specifically, a higher frequency of non-organizational R/S was associated with lower cognitive scores, while a higher frequency of religious and spiritual coping was associated with better cognitive scores in the whole sample. It is important to note that while higher non-organizational R/S was associated with lower cognitive scores, this does not imply causation. Individuals experiencing cognitive decline may turn more to private religious practices for support, thus inflating the association. These findings inform the ongoing investigation into the relationship between religious and spiritual facets and cognitive health and raise several important points for discussion.

### 4.1. Overall Sample Findings

These findings differ from other studies. Britt et al. (2022) reported higher frequency of private prayer alone was associated with better Global cognitive scores measured with the Clinical Dementia Rating (CDR) (Hughes et al. 1982; Morris 1993) across a small U.S. sample of participants living with dementia (mostly White participants (73.9%)). Koenig et al. (2004) reported that a higher frequency of private prayer (other than mealtimes) was also associated with better cognitive function with the Mini-Mental State Examination (MMSE) (Folstein et al. 1975; Koenig 1996) among hospitalized older adults in North Carolina, U.S., in a primarily white sample (61.2%). However, neither Britt et al. (2022), Koenig et al. (2004), nor our study (regarding non-organization R/S) identified which prayer *type* individuals were using. Laird et al. (2004) suggest there are five *types* of prayers: adoration (worshipping God or the sacred), confession (admitting wrong things done to God or the sacred), thanksgiving (offering thanks for specific things to God or the sacred), supplication (making specific requests to God or the sacred), and reception (opening oneself up to receive guidance and wisdom from God or the sacred). Differentiating the type of prayer in these associations could shed further light on findings. Since our study is cross-sectional, it is not possible to determine the temporal relationship. Our study suggests that individuals experiencing worse cognitive function may turn to prayer and reading for coping with these changes.

In addition, our study of non-organizational R/S included prayer frequency and reading religious and spiritual materials (i.e., books, magazines), which together may not support cognitive health as much as prayer alone. Older adults experiencing cognitive decline may avoid reading if it becomes more challenging; therefore, longitudinal studies are needed to examine these associations further. Chang et al. (2020) reported that reading activity in general may be protective of cognitive function in later life, but perhaps it depends on cognitive processing style. Reading religious materials (i.e., repeated and patterned religious activities) may support an automatic/intuitive cognitive processing style as opposed to a controlled/analytical processing style, potentially constraining cognitive stimulation as suggested by Hill et al. (2020), which would suggest that reading materials that prompt analytical cognitive processing are more cognitively beneficial (Nyborg 2009; Pennycook et al. 2016; Shenhav et al. 2012; Sherkat 2010; Vance 2004). This distinction between intuitive and analytical cognitive processing styles may partially explain the observed results. Repeated engagement with familiar religious or spiritual texts may stimulate automatic recall rather than new learning, which may limit cognitive stimulation over time, particularly in comparison to secular reading material that challenges reasoning or introduces novel content. In addition, it is also possible that lower cognitive scores influence reading and praying activities. Future studies could explore prayer frequency and reading religious and spiritual materials separately.

Our finding that a higher frequency of religious and spiritual coping was associated with better cognitive scores aligns with other studies. Koenig et al. (1992) found that religious and spiritual coping was positively correlated with better cognitive function in U.S. hospitalized male veterans in North Carolina (71.2% white sample). Religious and spiritual coping may lead to better cognitive functioning, or perhaps better cognitive function may facilitate religious and spiritual coping, considering the cognitive nature of these processes (Koenig et al. 2004). Researchers found that higher religious coping was associated with better cognitive function among U.S. nursing home residents based at the Veterans Affairs Medical Center in North Carolina (82.6% white sample) (Koenig et al. 1997). Interestingly, Vitorino et al. (2023) found that positive religious and spiritual coping in older adults in Southern Brazil was associated with better cognitive outcomes and a slower rate of cognitive decline over four years, as well; on the flip side, negative religious and spiritual coping was associated with poorer cognitive outcomes. Religious and spiritual coping describes behaviors, beliefs, and strategies individuals use to overcome stressors or difficult life circumstances (Pargament 2011). Positive religious and spiritual coping refers to a confident and trusting connection with God or the sacred (Hebert et al. 2009), while negative religious and spiritual coping refers to spiritual struggles, discontent, reflecting a less secure connection with God or the sacred (Hebert et al. 2009; Park et al. 2017). Our findings align with a higher and more positive religious and spiritual coping as diverse older adults responded to their connection to God and security in their religious and spiritual beliefs (Lukwago et al. 2001). These findings are similar to our more diverse sample with earlier studies in primarily white and Spanish-speaking samples mentioned above.

We did not find an association between religious and spiritual healing and cognitive health in the overall sample. In our study, religious and spiritual healing was operationalized through a single self-report item measuring frequency of prayer when ill, rather than broader healing practices such as meditation, guided imagery, or therapeutic spiritual counselling. This item of religious and spiritual healing (i.e., “when I am ill, I pray for healing”) appears to align more with supplication prayer—asking for help from God or the sacred rather than engaging in deeper religious and spiritual practices that may have cognitive benefits (e.g., meditation, mindfulness, gratitude-based prayer). It appears that religious and spiritual healing, as supplication prayer, is not associated with cognitive health based on our findings in diverse older adults; indeed, different types of prayers (e.g., contemplative vs. petitionary) may have different psychological and physiological effects (Ladd and Spilka 2002).

### 4.2. Gender-Specific Discussion

Our gender-specific analysis revealed that women, rather than men, primarily drove the observed associations. Higher non-organizational R/S and religious and spiritual healing were significantly associated with lower cognitive scores, while religious and spiritual coping maintained a positive association with cognitive scores for women. We did not identify any significant R/S associations in men. These findings differ from those (Ahrenfeldt et al. (2024), who found associations between higher frequency of religious attendance and better cognitive function in both European men and women, as well as higher frequency of prayer and better cognitive function in European men alone but not women. Women may tend more towards R/S and engage in religious and spiritual practices compared to men (Hvidtjørn et al. 2014; Munoz et al. 2015; Rassoulian et al. 2021). Factors influencing gender differences have included potential biological, societal, or general factors. Various religions dictate socially constructed gendered roles, norms, incentives, and expectations that may vary across religion and/or denomination (Maselko and Kubzansky 2006; Schnabel et al. 2018). Women scored higher than men on a National Institutes of Health Healing Experiences in All Life Stressors (NIH HEALS) tool measuring connection, reflection, and introspection, suggesting that women may have a higher self-reported psycho-social-spiritual healing perspective (Luna et al. 2019).

This expands our understanding that not only do women tend to be more religious and spiritual compared to men, engaging in religious and spiritual activities, but that perhaps cognitive health associations are supported by religious and spiritual activities in women alone. While the gender-stratified analysis suggests stronger associations among women, formal interaction tests could provide additional confirmation. Nevertheless, these findings support the development of gender-sensitive cognitive health strategies that leverage the spiritual resources often accessed by older women. Additional research is needed over time, examining a larger sample of diverse women to contribute additional understanding.

### 4.3. Race-Specific Discussion

Our race-specific analysis revealed some similarities and differences in associations across race. Higher religious and spiritual coping was associated with better cognitive scores in Black and White adults, while higher religious and spiritual healing was associated with worse cognitive scores in Black adults alone. No significant associations were identified for non-organizational R/S in either group.

Our results on the R/S facet of religious and spiritual coping is similar to Henderson et al. (2021) who reported that greater religiosity (i.e., capturing religious beliefs, values) was associated with better cognitive functioning among Black women but differed from ours in that we identified significant associations in White adults, too—Henderson et al. 2021) reported lower cognitive functioning associations with religiosity in White men and women. Indeed, our studies support the finding that both Black and White adults, and women alone, have a positive association between religious and spiritual coping and cognitive health. This adds consideration for including religious and spiritual coping in studies amongst Black and White women.

We found worse cognitive function for religious and spiritual healing among Black adults only, while Sauerteig-Rolston et al. (2024) found no significant associations for religious service attendance or religious salience with cognitive functioning among Black adults. Henderson et al. (2021) reported a similar negative association with religiosity, but for White adults only. Religious and spiritual healing in our study measured how often adults prayed for healing when they were ill. Black adults may rely more on prayer for improving their health compared to White adults. Overall health care use by White adults exceeds that of Black adults (Dickman et al. 2022), suggesting Black adults may face greater challenges in accessing providers and healthcare related to financial constraints, insurance barriers, and finding culturally competent providers (Key Data on Health and Health Care by Race and Ethnicity 2024). More research is needed to tease out this relationship, as many factors may underlie this association.

### 4.4. Implications

Findings from this study can inform culturally sensitive cognitive health programs that integrate religious and spiritual coping strategies, particularly for older Black and White women, who may benefit most. Faith-based organizations may also be strategic partners in promoting cognitive health in aging populations.

### 4.5. Strengths and Limitations

Our study was limited to the greater Philadelphia, Pennsylvania area in the Northeastern U.S. These findings were examined at one time point. Future studies are needed using a larger, nationally representative sample of the U.S. and over time to examine what religious and spiritual facets may be protective and which ones may not. In addition to examining associations in historically underrepresented groups at greater risk of MCI and AD/ADRD, future studies should examine associations in rural populations who are also at greater risk of MCI and AD/ADRD, yet no studies have focused on rural factors in spiritual practice associations with cognitive health. We used validated measures for cognitive function. To tease out additional components of religious and spiritual facets, we examined three religious and spiritual dimensions. Future studies should include validated measures for specific religious and spiritual components—not just religiosity. Additional religious and spiritual activities, such as meditation, meaning, and purpose, could further elucidate our understanding of the lifestyle factors of R/S that could influence cognitive health. In addition, our study is limited by recall bias as self-reported items were used in the religious and spiritual measure. Determining the effects of facets of R/S on cognitive health in our study is therefore not possible.

## 5. Conclusions

Our study sheds light on the utilization of religious and spiritual resources by diverse older adults in cognitive health. It appears women drove the significant findings, as religious and spiritual facets were significantly associated with cognitive health in women but not men. It appears that facets of R/S associations vary across race/ethnicity and gender. These findings provide additional evidence for spirituality and cognitive function, gender, and racial differences in a diverse sample, but additional studies are needed to tease out these differences and to inform future strategies to explore the delay of cognitive decline.

## Figures and Tables

**Table 1.** Descriptive Statistics of Sample (*N* = 1041).

	Gender		
	Men	Women	Total
***N***	459 (44.1%)	582 (55.9%)	1041 (100.0%)
Total MoCA Score	24.906 (3.731)	25.069 (4.186)	24.997 (3.991)
Non-organizational R/S	10.597 (12.253)	12.266 (15.115)	11.530 (13.944)
Religious & Spiritual Coping	9.044 (9.293)	10.282 (11.303)	9.736 (10.478)
Religious & Spiritual Healing	3.000 (3.112)	3.424 (3.786)	3.237 (3.509)
**Race**			
Black	239 (52.1%)	334 (57.4%)	573 (55.0%)
White	220 (47.9%)	248 (42.6%)	468 (45.0%)
Age	68.802 (8.792)	68.358 (9.136)	68.554 (8.985)
**Educational level**	4.083 (1.489)	3.978 (1.438)	4.024 (1.461)
**Financial Constraints**			
Some $ left	286 (62.3%)	362 (62.2%)	648 (62.2%)
Just enough	111 (24.2%)	145 (24.9%)	256 (24.6%)
Not enough	62 (13.5%)	75 (12.9%)	137 (13.2%)
**Multiple Chronic Conditions**			
No	63 (13.7%)	57 (9.8%)	120 (11.5%)
Yes	396 (86.3%)	525 (90.2%)	921 (88.5%)

*Notes.* MoCA = Montreal Cognitive Assessment.

**Table 2.** Regression Models for Association Between Dimensions of Religion/Spirituality (R/S) and MoCA Score.

	Model 1	Model 2
	
	Unadjusted	Adjusted
Non-organizational R/S	−0.240 ***	−0.153 **
	(0.059)	(0.05)
Religious & Spiritual Coping	0.402 ***	0.278 ***
	(0.089)	(0.076)
Religious & Spiritual Healing	−0.289	−0.177
	(0.227)	(0.192)
Race (Ref: White)		
Black		−2.333 ***
		(0.242)
Age		−0.099 ***
		(0.012)
Gender (Ref: Men)		
Women		0.292
		(0.209)
Educational Level		0.714 ***
Financial Constraints		(0.084)
Some $ Left		0.846 **
		(0.337)
Just Enough		0.091
		(0.357)
Multiple Chronic Conditions (Ref: No)		
Yes		0.298
		(0.334)
Constant	24.78 ***	28.87 ***
	(0.184)	(0.98)
Observations	1041	1041
R-squared	0.023	0.308
Prob > F	0.000	0.000
F-test	8.131	45.784
Akaike crit. (AIC)	5818.46	5473.81
Bayesian crit. (BIC)	5838.25	5528.24

Standard errors in parentheses.

*** *p* < 0.01

** *p* < 0.05

* *p* < 0.1.

**Table 3.** Regression Models for Association Between Dimensions of Religion/Spirituality (R/S) and MoCA Score Stratified by Gender.

	Men	Women
Non-organizational R/S	−0.036	−0.17 **
	(0.073)	(0.076)
Religious & Spiritual Coping	0.208	0.454 ***
	(0.111)	(0.104)
Religious & Spiritual Healing	−0.216	−0.32 **
	(0.167)	(0.137)
Race (Ref: White)		
Black	−1.861 ***	−2.526 ***
	(0.365)	(0.325)
Age	−0.071 ***	−0.112 ***
	(0.019)	(0.016)
Educational Level	0.665 ***	0.703 ***
Financial Constraints	(0.126)	(0.113)
Some $ Left	0.173	1.225 ***
	(0.495)	(0.459)
Just Enough	−0.526	0.526
	(0.533)	(0.479)
Multiple Chronic Conditions (Ref: No)		
Yes	0.282	0.274
	(0.46)	(0.484)
Constant	27.546 ***	29.717 ***
	(1.456)	(1.304)
Observations	459	582
R-squared	0.231	0.376
Prob > F	0.000	0.000
F-test	14.971	38.239
Akaike crit. (AIC)	2409.801	3063.086
Bayesian crit. (BIC)	2451.091	3106.751

Standard errors in parentheses.

*** *p* < 0.01

** *p* < 0.05.

**Table 4.** Regression Models for Association Between Dimensions of Religion/Spirituality (R/S) and MoCA Score Stratified by Race.

	Black	White
Non-organizational R/S	−0.123	−0.139
	(0.084)	(0.075)
Religious & Spiritual Coping	0.443 ***	0.226 ***
	(0.124)	(0.085)
Religious & Spiritual Healing	−0.393 **	−0.223
	(0.167)	(0.127)
Age	−0.085 ***	−0.11 ***
	(0.018)	(0.016)
Gender (Ref: Men)		
Women	−0.151	0.806 ***
	(0.317)	(0.269)
Educational Level	0.872 ***	0.413 ***
	(0.125)	(0.109)
Financial Constraints		
Some $ Left	0.638	0.815
	(0.44)	(0.556)
Just Enough	0.157	−0.042
	(0.454)	(0.614)
Multiple Chronic Conditions (Ref: No)		
Yes	0.52	0.268
	(0.644)	(0.344)
Constant	25.136 ***	31.755 ***
	(1.402)	(1.358)
Observations	573	468
R-squared	0.173	0.216
Prob > F	0.000	0.000
F-test	13.040	14.034
Akaike crit. (AIC)	3134.180	2293.454
Bayesian crit. (BIC)	3177.689	2334.939

Standard errors in parentheses.

*** *p* < 0.01

** *p* < 0.05.

## Data Availability

The data supporting the conclusions of this article will be made available by the authors on request.

## Abbreviations

R/S: Religion/Spirituality
MCI: Mild cognitive impairment
AD: Alzheimer’s disease
ADRD: Alzheimer’s disease related dementias
